# Experimental manipulation of immune-mediated disease and its fitness costs for rodent malaria parasites

**DOI:** 10.1186/1471-2148-8-128

**Published:** 2008-04-30

**Authors:** Gráinne H Long, Brian HK Chan, Judith E Allen, Andrew F Read, Andrea L Graham

**Affiliations:** 1 Institutes of Evolution, Immunology and Infection Research, School of Biological Sciences, University of Edinburgh, King's Buildings, West Mains Road, Edinburgh, EH9 3JT, UK; 2 Center for Infectious Disease Dynamics, Department of Biology, Mueller Laboratory, The Pennsylvania State University, University Park, Pennsylvania 16802, USA

## Abstract

**Background:**

Explaining parasite virulence (harm to the host) represents a major challenge for evolutionary and biomedical scientists alike. Most theoretical models of virulence evolution assume that virulence arises as a direct consequence of host exploitation, the process whereby parasites convert host resources into transmission opportunities. However, infection-induced disease can be immune-mediated (immunopathology). Little is known about how immunopathology affects parasite fitness, or how it will affect the evolution of parasite virulence. Here we studied the effects of immunopathology on infection-induced host mortality rate and lifetime transmission potential – key components of parasite fitness – using the rodent malaria model, *Plasmodium chabaudi chabaudi*.

**Results:**

Neutralizing interleukin [IL]-10, an important regulator of inflammation, allowed us to experimentally increase the proportion of virulence due to immunopathology for eight parasite clones. *In vivo *blockade of the IL-10 receptor (IL-10R) with a neutralizing antibody resulted in a shorter time to death that was independent of parasite density and was particularly marked for normally avirulent clones. This suggests that IL-10 induction may provide a pathway to avirulence for *P. c. chabaudi*. Despite the increased investment in transmission-stage parasites observed for some clones in response to IL-10R blockade, experimental enhancement of immunopathology incurred a uniform fitness cost to all parasite clones by reducing lifetime transmission potential.

**Conclusion:**

This is the first experimental study to demonstrate that infection-induced immunopathology and parasite genetic variability may together have the potential to shape virulence evolution. In accord with recent theory, the data show that some forms of immunopathology may select for parasites that make hosts less sick.

## Background

Understanding why some pathogens are more virulent than others is a major challenge. Medical and veterinary science seldom look for evolutionary explanations of why virulent pathogen strains might spread in a host population, but recent theoretical advances suggest that an evolutionary approach could provide more effective ways to manage infectious disease [1]. Much current thinking on the evolution of parasite virulence, defined here as mortality due to infection, is based on the parasite-centric idea that virulence is an incidental and unavoidable consequence of extracting resources from hosts in order to generate transmission opportunities. Under this view, high levels of host exploitation enhance transmission (the fitness benefit of virulence), but increase the risk of host death (the fitness cost of virulence). This assumed association between virulence and transmission represents an evolutionary trade-off between how extensively parasites replicate and the duration of the infectious period. According to this trade-off view, parasite virulence is optimized by balancing virulence and replication such that transmission is maximized over the duration of infection [2-6].

Most theory on the evolution of virulence is therefore based on two key assumptions: that disease severity increases with parasite exploitation of hosts, and that immunity acts solely to reduce parasite titres and the probability of death. However, biomedical literature shows that a very significant proportion of infection-induced disease can be immune-mediated, and even independent of parasite replication. Indeed, many important human infectious diseases are most lethal when associated with immune-mediated disease (immunopathology), regardless of parasite density [7]. In malaria, for example, the host immune response has been implicated as the cause of many symptoms of infection [8-10] and may help to explain the discordance that is often observed between virulence and parasite load [11,12]. Immunological hyperactivity has also been shown to play a role in the pathogenesis of mucosal [13] and cutaneous leishmaniasis [14] as well as the H5N1 [15] and, most likely, 1918 influenza viruses [16], among other infections. An unregulated immune response to infection has the potential to cause substantial damage to host tissues.

Immune responses can thus kill both hosts and parasites. For the most part, evolutionary theory assumes only the latter, but even that is not always right: some immune responses cause disease without killing parasites. For example, in schistosomiasis, immune responses to parasite eggs can cause serious liver disease that is unrelated to the clearance of adult parasites [17]. Recognising this, several authors have argued that immunopathology is a major complication for the parasite-centric trade-off model of virulence evolution (e.g., [7,18,19]), and the implications of excessive immune responses for standard models of the evolution of virulence are now beginning to be subject to theoretical investigation (e.g., [20,21]). Indeed, recent theoretical work has shown that immunopathology has the potential to alter the cost/benefit ratio of virulence and may thus alter the trajectory of parasite virulence evolution [20,21]. A key empirical question which arises is how immunopathology affects parasite fitness. Do parasites experience increased or decreased transmission when hosts suffer immune-mediated disease? Does the induction of immunopathology, as well as its effect upon transmission, vary by parasite genotype? The answers to these questions would elucidate whether and how natural selection due to immunopathology could shape parasite virulence.

Here, we addressed these questions in the context of malaria, where it has been argued that the trade-off model applies [22]. We explored the effect of immunopathology on key fitness components of the parasite – infection-induced mortality and lifetime transmission potential – using the rodent malaria parasite, *Plasmodium chabaudi chabaudi*. In this system, a rapid pro-inflammatory response is essential to control rising parasitaemia, but an overzealous induction of the same mediators is associated with immunopathology and poor disease outcome [23,24]. To experimentally manipulate immunopathological virulence, we used a reagent to neutralize the activity of Interleukin [IL]-10, a potent anti-inflammatory molecule [25] which helps to prevent immune hyperactivity during malaria infection. Previous studies using rodent models of malaria infection have shown that ablating IL-10 signalling promotes the development of immune-mediated cerebral malaria [26,27] and results in lethal inflammatory responses [28-30]. By using IL-10 receptor-specific monoclonal antibodies (IL-10R mAb IgG, BD PharMingen, hereafter called αIL-10R) to block *in vivo *IL-10R signalling during rodent malaria infection [31], we experimentally generated an immune-mediated increase in host mortality rate. We then explored the genotype-specificity of this effect, as well as the consequences an increase in immunopathological virulence may have on the number of transmission-stage parasites (gametocytes) produced.

## Results

### IL-10 receptor blockade differentially decreased time to death, depending on parasite genotype

**Figure 1:**
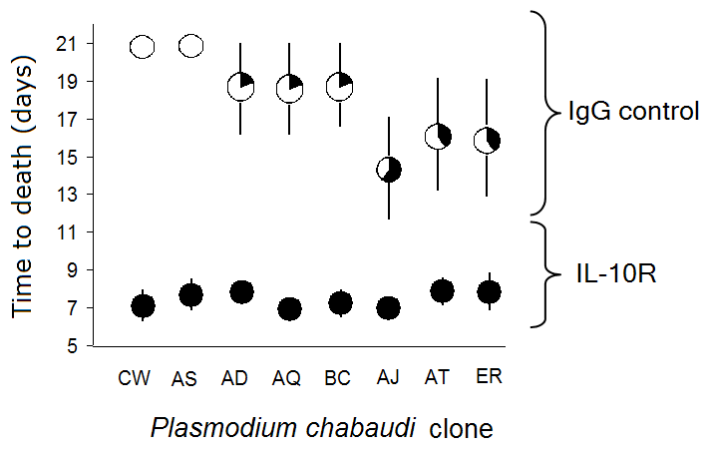
**Effect of IL-10R blockade on the extent decreased survival time depended on *P. c. chabaudi *parasite genotype**. C57BL/6J mice were administered neutralizing αIL-10R mAb or control IgG, 1 day before and on days 1, 2, 3 and 4 post infection with 10^6 ^parasites of one of eight distinct *P. c. chabaudi *clones (CW, AS, AD, AQ, BC, AJ, AT or ER). Each pie-chart symbol represents the proportion of mice surviving until day 21 (white fill), while the position of the pie-chart along the y-axis indicates mean time to death in that treatment group (based on the mean of five mice ± S.E.). Day 21 was chosen as the end-point, to ensure that the acute phase of malaria infection was captured. Thus a mean of 21 without variance and with solid white fill indicates no mice died from that treatment group. Relative to control mice, αIL-10R treated mice suffered a higher mortality rate (~15% versus ~100%, respectively) and a quicker time to death (average time to death: 18.1 +/- 0.1 versus 7.5+/-0.2 days, respectively) and normally avirulent *P. c. chabaudi *clones were brought to lethality when IL-10R was blocked.

IL-10R blockade significantly decreased the survival of infected mice (Figure 1; treatment: *Z *= 2.02, *P *= 0.04, n = 80). For example, 100% of AD-infected mice died before day 9 p.i. if IL-10 signalling was blocked, whereas only 20% of control AD mice died by day 21 (Figure 1). The extent to which IL-10R blockade affected the time to death depended on parasite genotype (Figure 1; clone: χ^2^_7, _= 18.56, *P *= 0.01, treatment × clone: χ^2^_7, _= 23.05, *P *= 0.002): for example, the increase in immunopathological virulence was greatest for the normally avirulent clones CW or AS, which were brought to lethality (Bonferroni *P *≤ 0.025 for pair-wise comparisons of the IgG versus αIL-10R treatments for clones CW or AS; *P *≥ 0.09 for all other pair-wise comparisons of treatment effects). Thus, IL-10 R blockade pushed normally benign infections to lethality.

### Total parasite burdens did not explain the increase in mortality

**Figure 2:**
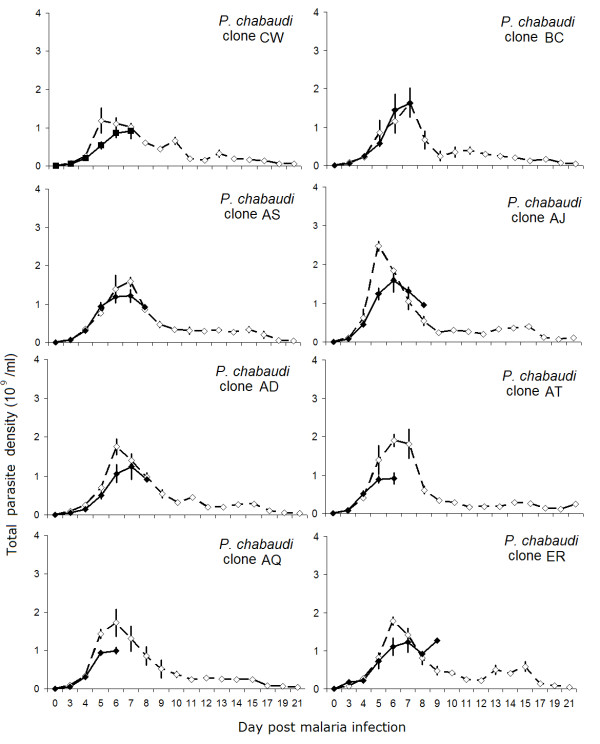
**Total parasite densities were lower during IL-10R blockade, regardless of *P. c. chabaudi *parasite genotype**. Line graphs represent the dynamics of total parasite density in αIL-10R (filled symbols) or control IgG (open symbols) treated mice during single-clone infections with each of eight *P. c. chabaudi *clones. Each line represents the mean of five mice (± S.E.), except where deaths had occurred. Regardless of *P. c. chabaudi *parasite genotype, neutralizing IL-10R resulted in lower total parasite densities between days 3 to 21 p.i. and lower early parasite densities (days 3–8 p.i.) compared to control mice.

**Figure 3:**
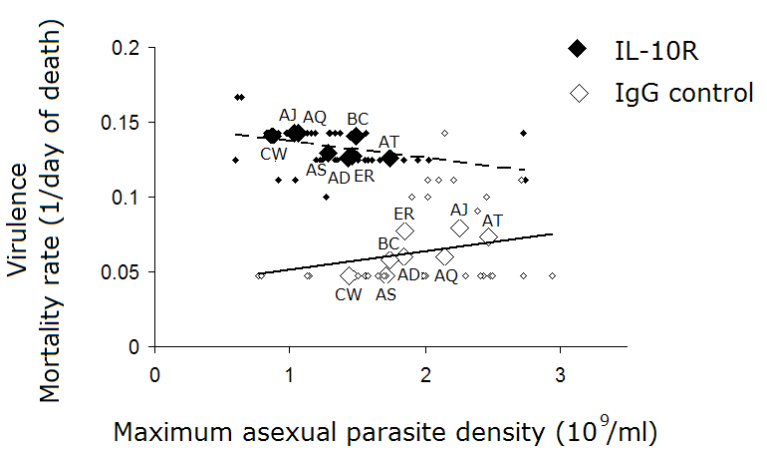
**Effect of IL-10R blockade on the relationship between virulence and asexual parasite density**. Virulence, as measured in terms of mortality rate (1/day of death), is plotted against maximum parasite density for each of 8 parasite genotypes in αIL-10R (filled symbols) versus IgG control (open symbols) treated mice. Pearson correlation analyses revealed a significant negative relationship between virulence and asexual parasite density when IL-10R was blocked, but not in control mice. This suggests that the αIL-10R-driven increase in mortality was caused by uncontrolled inflammatory responses, and not increases in parasite load. Indeed, the same pro-inflammatory molecules that kill parasites also kill hosts [29]. Each large symbol represents the mean of five mice (± S.E) and small symbols represent individual mice.

Mice with IL-10R blockade had significantly lower total and peak parasite densities compared to control mice (Figure 2; treatment *F*_1,64 _= 192.9, *P *< 0.0001; treatment *F*_1,64 _= 41.8, *P *< 0.0001, respectively). Clones differed in total and peak parasite density, in agreement with previous work [32], but the effect of IL-10R blockade on parasite load did not vary across clones (Figure 2; for total parasite density, clone: *F*_7,64 _= 2.69, *P *= 0.017; and treatment × clone: *F*_7,64 _= 1.1, *P *= 0.39; for peak parasite density, clone: *F*_7,64 _= 3.53, *P *= 0.003; and treatment × clone: *F*_7,64 _= 1.6, *P *= 0.15). In addition, mortality rates decreased with increasing peak parasite density in the IL-10R treated group, but not in the control group (Figure 3: r = -0.34, *P *= 0.01 and r = 0.22, *P *= 0.17, respectively). The same pattern was observed for total parasite density (data not shown). These data suggest that an uncontrolled inflammatory response, and not increased parasite load, caused the increase in mortality following IL-10R blockade.

### IL-10R blockade altered the transmission potential of all parasite genotypes

**Figure 4:**
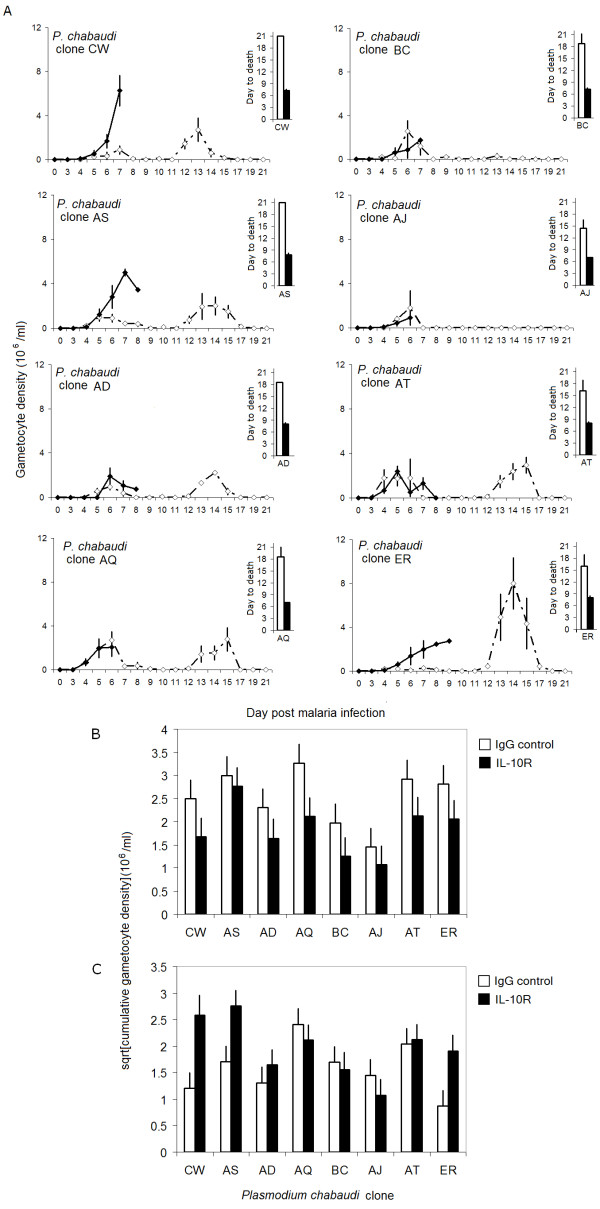
**The mortality caused by IL-10R blockade incurred a universal cost to lifetime transmission potential, but early in infection clones differed in their response to IL-10R blockade**. (A) Line graphs compare the dynamics of gametocyte density during IL-10R blockade (filled symbols) or control IgG treatment (open symbols) during single-clone infections with each of eight *P. c. chabaudi *clones. Insets show day to death for each of the clones in αIL-10R (filled bars) versus IgG (open bars) treated mice. Bar graphs represent the least squares mean of total gametocyte density (days 4–21 p.i. inclusive, an estimate of lifetime transmission potential) (B), or early gametocyte density (days 3–8 p.i. inclusive) (C), broken down by treatment and *P. c. chabaudi *parasite clone. Each line or bar represents the mean of five mice (± S.E.), except where deaths had occurred. Regardless of *P. c. chabaudi *parasite genotype, blocking IL-10R reduced the lifetime transmission potential relative to control mice (A and B). However, the extent to which IL-10R blockade directly affected early gametocyte density depended on *P. c. chabaudi *parasite clone (C).

The total gametocyte density produced during infection was significantly lower in mice with IL-10R blockade, regardless of parasite genotype (Figure 4A and 4B; treatment: *F*_1,60 _= 8.8, *P *= 0.004, clone: *F*_7,60 _= 3.85, *P *= 0.002, treatment × clone: *F*_7,60 _= 0.47, *P *= 0.85). Taking day of death into account allowed us to test statistically whether the immune-mediated increase in mortality caused by IL-10R blockade (see Figure 1 and day to death insets in Figure 4A) incurred a cost to the lifetime transmission potential. Statistically controlling for day to death (*F*_1,59 _= 7.14, *P *= 0.01) accounted for the effects of IL-10R blockade on lifetime transmission potential (treatment: *F*_1,59 _= 0.56, *P *= 0.46, clone: *F*_7,59 _= 3.85, *P *= 0.002, treatment × clone: *F*_7,59 _= 0.74, *P *= 0.64). Furthermore, total parasite load did not significantly predict lifetime transmission potential (*F*_1,58 _= 0.54, *P *= 0.47) and including it in the above statistical models did not alter the effects of IL-10R blockade or parasite genotype (data not shown). These data suggest that the immune-mediated increase in mortality rate caused by blocking IL-10R imposed a fitness cost to the parasite in terms of decreased lifetime transmission potential regardless of parasite genotype and independent of total parasite load. As total gametocyte density should closely correlate with parasite fitness in this system [32-34], these data suggest that the increase in immune-mediated mortality incurred a universal cost to parasite fitness.

However, the total gametocyte density reached before day 8 post infection (total early gametocyte density) of distinct clones was differentially affected by treatment (Figure. 4C; treatment: *F*_1,60 _= 5.4, *P *= 0.024, clone: *F*_7,60 _= 4, *P *= 0.001, treatment × clone: *F*_7,60 _= 3.3, *P *= 0.005). In particular, IL-10R blockade tended to increase the early transmission potential of clones CW, AS or ER (Figure. 4A and 4C: Bonferroni-corrected *P *= 0.025, *P *= 0.070 and *P *= 0.077, respectively) but not for the remaining clones (Bonferroni *P *= 0.2 for treatment within clone). These results held even when parasite load during this early time period was controlled for (data not shown). Thus, early in infection (i.e., before αL-10R treatment caused death), the effect of IL-10R blockade on gametocyte density depended on parasite genotype; being beneficial to the transmission potential of clones CW, AS and ER, while not affecting the transmission potential of clones AT, AQ, BC, AJ and AD.

## Discussion

The role pathological immune responses play in shaping the evolution of parasite virulence is an important question that has not previously been empirically addressed. In this study, we examined how immunopathological virulence affects the fitness of rodent malaria parasites. We experimentally manipulated the potent anti-inflammatory cytokine IL-10 [25,35], an immune signaling molecule that is thought to be under balancing selection in human populations: genetic predisposition to strong IL-10 responses is beneficial in terms of avoiding excessive inflammation but costly in terms of reduced ability of hosts to control parasite replication [36]. In rodent models of infectious disease, neutralizing IL-10 leads to uncontrolled pro-inflammatory responses and thus increased immunopathological virulence of many parasites [37-39]. Indeed, the importance of IL-10 in conferring protection against lethal inflammatory responses during acute *P. c. chabaudi *infection with a single parasite genotype (clone AS) is well established [27-30]. However, the effect of overzealous immune responses on the lifetime transmission potential of genetically distinct malaria parasites that vary in virulence has not, until now, been examined.

We found that IL-10R blockade increased the risk of host death, independent of the total parasite burden. Indeed, in that treatment group, mortality rate actually increased with decreasing parasite density (Figure 3). This result is unsurprising because the same molecules that promote malaria parasite clearance also cause direct harm to the host – for example, cerebral hemorrhage and edema [27]. All infections were brought to lethality when IL-10R was blocked, but this effect was most pronounced for the normally benign clones CW and AS, which were converted from avirulent to killer parasites (Figure 1). It may be that their characteristic avirulence [32] is related in part to their induction of anti-inflammatory molecules such as IL-10 (discussed in more detail below). We also showed that the immunopathological acceleration of host death imposed a universal fitness cost to the parasites, in terms of reduced lifetime transmission potential. Early in infection, however, some genotypes generated more gametocytes in IL-10R neutralized hosts. These results suggest that immunopathological virulence has the potential to strongly affect parasite fitness, both indirectly (via host death) and directly (via alterations in the production or survival of the gametocytes themselves).

The classical adaptive trade-off hypothesis posits that host immunity may select for higher levels of virulence [40-42]. This theory has found empirical support in both rodent malaria and myxomatosis [43,44]. For example, previous work on *P. c. chabaudi *in mice revealed that the induction of an immune response which clears parasites preserves positive virulence-transmission relationships [45] and promotes the evolution of greater virulence [46]. In Australian rabbits, myxomatosis initially caused a case fatality rate of ~99.9%, quickly evolved to intermediate levels of virulence [47], and later become more virulent [48]. Selection for enhanced resistance (i.e., immunological control of the virus) has been implicated in driving myxoma virulence upwards again [49,50]. Thus, there is empirical support for the theory that protective immune responses select for more virulent parasites.

However, whether the predictions of classical theory hold in the face of pathogenic immune responses, and thus an immune-mediated component to parasite virulence, will depend on how immunopathology affects lifetime transmission potential. Recent theoretical developments confirm that immunopathology is likely to alter the trajectory of virulence evolution in certain contexts [21]. Essentially, the mechanism by which immune-mediated disease arises matters. For example, if immunopathology arises independently of parasite exploitation (estimated here as parasite density), evolution is expected to favor increased virulence. In that case, immunopathology has the effect of increased background mortality; regardless of what the parasite does, the host will die, so it should aggressively exploit its host. On the other hand, if immunopathology increases with increased host exploitation – particularly when the immunopathological "side effect" of exploitation is greater for higher rates of immune clearance – then parasites which more prudently exploit their hosts would be favored. In such cases, more benign parasite strains will spread through the host population [21]. For *P. c. chabaudi *in immunologically intact animals, we expect immunopathology to increase with exploitation, because autoimmune destruction of uninfected red blood cells, for example, scales proportionally with parasite density in this system [51]. At the same time, our empirical results indicate that experimentally-induced increases in inflammation (a likely correlate of clearance rate) increase virulence (Figure 1), and that virulent parasite genotypes induce more inflammation per parasite, in unmanipulated hosts [52]. Thus, for *P. c. chabaudi*, the immunopathological side effect of exploitation may indeed be higher for higher rates of immune clearance. Together, these observations suggest that evolution might favor reduced malaria virulence [21]. However, protective immune responses against malaria impose the opposite selection pressure [46], and the relative strengths of these potentially conflicting effects of immunity remain to be investigated. Regardless of the exact mechanism, it appears that expanding the trade-off theory to incorporate immunopathology [21] may be important for understanding the evolution of malaria parasites.

Differences among parasite genotypes in the extent to which their characteristic virulence appears to depend upon IL-10 induction are also highlighted by our results. The panel of eight clones represents a spectrum of genetic diversity for virulence [32]. Although clone virulence does increase with increasing parasite densities overall [32], there is variation about this relationship which may be caused by differential propensity to modulate pathological immune responses. We have previously shown that a subset of the clones varies in per-parasite virulence as well as the immune responses that they induce [52], as outlined above. Clone BC, for example, induces strong pro-inflammatory responses and causes high virulence at relatively low parasite densities, whereas the avirulent clones AS and CW induce significantly higher plasma concentrations of IL-10 and lower concentrations of pro-inflammatory molecules compared to virulent clones [52]. In support of these associations, the virulence of AS and CW in the present study was highly sensitive to the absence of IL-10 signalling (Figure 1), though we acknowledge that our experimental manipulation may have maximized virulence and contributed to the lack of clone differences in mortality rate in that treatment group.

One approach to prudent exploitation could thus include avoidance of immunopathology via induction of anti-inflammatory factors such as IL-10. This could have a combined benefit for parasites: impairing both protective and pathological immune responses, thereby permitting high parasite densities while minimizing per-parasite virulence. Many extracellular pathogens are thought to exploit IL-10 in order to subvert host immunity and enhance parasite survival [53]. For example, both Epstein-Barr virus (EBV) [54] and cytomegalovirus [55] infected cells express a viral homologue of IL-10 with similar immunomodulatory activity to host IL-10. In addition, induction of IL-10 allows chronic lymphocytic choriomeningitis infection to persist, with IL-10R blockade resulting in viral clearance [56,57]. Immunopathology associated with these viruses may be ameliorated by IL-10 as well. If IL-10 mediated pathways are indeed proven to be a mechanism of avirulence during *P. c. chabaudi *infection, it suggests that immunomanipulation by parasites may function to control both protective (parasite-killing) and pathological host responses. Thus, for a given level of exploitation, selection might favor rodent malaria parasite genotypes that induce anti-inflammatory molecules. Of biomedical interest, it has been suggested that the cellular source of IL-10 may vary in response to infection with different pathogens (or even genetically distinct isolates of the same pathogen), the stage of infection, or anatomical location [25]. It would be interesting to investigate whether differences among *P. c. chabaudi *clones in the cellular source or timing of IL-10 induction play a role in determining the characteristic virulence schedules we observe.

Another potential role for parasite genotype in this system is raised by our observation that early in infection, IL-10R blockade directly and differentially affected the gametocyte load of distinct parasite genotypes (Figure 4A and 4C). The absence of IL-10 signalling was beneficial to the short-term transmission potential of clones CW, AS and ER but did not significantly affect clones AT, AQ, BC, AJ and AD (Figure 4C), even when asexual parasite load was taken into account. It is possible that the gametocytes of different clones are differentially sensitive to being killed by the immune system, though previous experiments have showed no differences in sensitivity to the pro-inflammatory molecule Tumor Necrosis Factor (TNF)-α for a subset of the clones studied here [58]. An alternative explanation for Figure 4C is related to the observation that rates of gametocyte production often increase under conditions which do not favour asexual replication [59-61]. For instance, the rate of gametocyte production during *P. c. chabaudi *infection increases during sub-curative anti-malarial chemotherapy [33,62]. Thus, it is possible that changes to the immune environment caused by manipulating IL-10 could trigger clones CW, AS and ER to increase gametocyte production. Why clones might differ in their cue for gametocytogenesis, and indeed whether transient increases in gametocyte density in response to excess inflammation matter evolutionarily, given the decrease in transmission potential caused by early host death, is unclear. The immune-mediated increase in early gametocyte density may indeed be important in natural populations in which hosts may suffer other sources of mortality during an infection, thereby placing a greater value on early gametocytogenesis. Theory [21] could possibly be extended to include cases where immunopathology directly affects transmission (as opposed to affecting transmission only via host death).

## Conclusion

Immune responses which kill parasites can have the side effect of killing hosts. This fact has important implications for host defense strategies, and may have shaped the evolution of the vertebrate immune system [7]. Here we have focused our discussion on parasite evolution. In our experiments, immunopathology reduced lifetime transmission potential of parasites by reducing host life expectancy. We also observed parasite genetic diversity in the extent to which the characteristic virulence of a clone depended upon IL-10. These observations together suggest the following possibilities. Selection favors parasite strains which effectively exploit hosts, but those strains may inevitably induce immune-mediated disease; one can thus imagine that selection should also favor strains able to modify the immunopathological consequences of infection. How the potentially conflicting selective pressures imposed by protective and pathological aspects of host immunity interact and play out will determine whether selection favors strains which cause more or less severe disease. In addition, our results imply that the evolutionary trajectory of malaria virulence may be altered by changes to the IL-10 milieu of a host population. A prevalent example in nature would be the presence of IL-10-inducing helminths in co-infected populations [63,64]. All of these hypotheses merit further investigation – for example, via experimental evolution of malaria parasites[46] in host lineages particularly prone to immunopathological virulence such as the αIL-10R treated hosts in the present study, versus those particularly unlikely to exhibit immunopathology such as hosts to whom recombinant IL-10 is experimentally added. Molecular-level manipulation of parasite phenotypes (e.g., in induction of inflammation) could also prove useful tools in studies of experimental evolution. More generally, our results lend support for detailed investigations of the relationship between immunopathological virulence and transmissibility in parasites of medical importance. Such studies could elucidate the consequences of not only lethal but also sublethal immunopathologies for parasite transmission (e.g., including the effects of fever itself [10] and/or host debilitation [4] on transmission biology). We contend that a better understanding of how host immunity – both protective and pathological – affects parasite transmission in the field would help shed light on the limits to and causes of parasite virulence in nature.

## Methods

### Mice and parasites

Mice were 6–8 week old female inbred C57BL/6 mice (bred in house). *Plasmodium chabaudi chabaudi *(*P. c. chabaudi*: [65]) clones were derived from African thicket rat isolates and stored as frozen stabilites [66]. Eight clones with different virulence schedules were used [32], with subscripts denoting their precise clonal histories; CW_706_, AS_12280_, AD_119_, AQ_231_, BC_248_, AJ_5166_, AT_89 _and ER_641 _(herein referred to as clones CW, AS, AD, AQ, BC, AJ, AT and ER). *P. c. chabaudi *AS infection is normally non-lethal in the strain of mice we used (C57BL/6J) [67] and previous studies from our lab have shown a proportion of sub-lethal virulence (e.g., anaemia or loss of body mass) is immune-mediated and independent of parasite density [52]. Mice were inoculated and housed as described previously [52].

### IL-10R blockade

IL-10 signalling can be neutralized *in vivo *during rodent malaria infections by the intra peritoneal (i.p.) injection of 20 μg of neutralizing anti-IL-10R monoclonal IgG antibody (clone 1B1.3a; BD PharMingen) [31]. Using this procedure, groups of mice were injected i.p. with control IgG (Sigma-Aldrich) or αIL-10R and, the following day, single-clone infections were initiated as described previously, at 10^6 ^parasite dose ([52], 5 mice per *P. c. chabaudi *clone or uninfected control group, per antibody treatment: n = 100 mice). Antibody treatments were applied again on days 1 to 4 post-infection inclusively. The uninfected but IL-10 depleted group experienced no mortality, nor loss of red blood cell density or body mass, so IL-10R treatment *per se *does not promote virulence in the absence of infection.

### Parasite density and virulence

Thin blood smears prepared from tail blood were Giemsa stained and microscopically analysed (× 1000 magnification) to calculate the proportion of RBCs parasitized with gametocytes and asexual parasites. Each day post infection, RBC densities were obtained by flow cytometery (Beckmann Coulter) by standard methods [52]. Daily gametocyte and parasite densities (per ml blood) were calculated by multiplying each corresponding proportion by the appropriate RBC density. Time to death was recorded until the experiment was terminated on day 21 p.i.

### Trait definition

Mortality data were analysed as time to death (days). Parasite load was quantified as the total parasite density reached by day 21 p.i. and, as the vast majority of deaths occurred before day 8 (~80%), data were also analysed as the total parasite load reached by day 8 p.i. (before the majority of deaths occurred). Gametocyte density correlates positively with infectivity to mosquitoes and, when summed over course of infection, provides an indicator of lifetime transmission potential [32-34]. Lifetime transmission potential was quantified as the total gametocyte density produced throughout the infection (days 3–21 p.i. inclusive). The total gametocyte density reached before day 8 post infection (total early gametocyte density) was also analyzed. Four parasitized mice failed to develop gametocytes and were not included in our analyses of gametocyte data. Prior to statistical analysis the following transformations were applied to meet the necessary normality and homogeneity-of-variances assumptions: square root (gametocyte and parasite density) and 1/square root(time to death).

### Statistical analysis

All parasite and gametocyte data were analysed using ANOVAs and ANCOVAs in Minitab (v. 14, Minitab Inc.). Time-to-death data were analysed using Minitab's "regression with life data" command which uses a modified Newton-Raphson algorithm to calculate maximum likelihood estimates of model parameters. "Regression with life data" accepts right censored data and a Weibull distribution was used in our models. The analysis is based upon a Z statistic for factors with 1 degree of freedom. For predictor variables with more than two levels (e.g., clone), significance tests were based upon assessment of improvement in model fit using a χ^2 ^distribution. For all analyses, explanatory variables used were antibody 'treatment' (anti-IL-10R or hu-IgG), infecting *P. c. chabaudi *'clone' (CW, AS, AD, AQ, BC, AJ, AT and ER) and the interaction between treatment and clone. To determine whether treatment or clone had effects on time to death and gametocytes beyond those exerted via total parasite load, data were analysed with and without total parasite load as a covariate. The maximal model was first fit to the data including treatment, clone, an interaction between treatment and clone, and covariate when relevant. Models were minimized by removing non-significant terms (P > 0.05), beginning with the interaction. The relationship between mortality rate (1/day of death) and asexual parasite density was analysed for all mice within a treatment using Pearson's correlation coefficient.

## Authors' contributions

GHL, AFR and ALG conceived the study and, together with JEA, designed the experiments, conducted statistical analysis and wrote the manuscript. BHKC coordinated and carried out animal handling, and GHL collected the data. All authors read and approved the final manuscript.
